# Comparison of Tricuspid Annular Plane Systolic Excursion to Pulmonary Artery Systolic Pressure Ratio Measured by Transthoracic Echocardiography and Right Heart Catheterization in Pulmonary Arterial Hypertension: Prognostic Implications

**DOI:** 10.3390/jcm14061968

**Published:** 2025-03-14

**Authors:** Eva Gutiérrez-Ortiz, Carmen Jiménez López-Guarch, Juan Francisco Delgado Jiménez, María Lorena Coronel, Irene Martín de Miguel, Belen Biscotti Rodil, Juan Duarte Torres, Teresa Segura de la Cal, María Teresa Velázquez Martín, Fernando Arribas Ynsaurriaga, Alejandro Cruz-Utrilla, Pilar Escribano-Subías

**Affiliations:** 1 Pulmonary Hypertension Unit, Department of Cardiology, Hospital Universitario 12 de Octubre, 28041 Madrid, Spain; egutierrezo@salud.madrid.org (E.G.-O.); cjimenez@salud.madrid.org (C.J.L.-G.); imdemiguel@salud.madrid.org (I.M.d.M.); belen.biscotti@salud.madrid.org (B.B.R.); mariateresa.velazquez@salud.madrid.org (M.T.V.M.); alejandro.cruz@salud.madrid.org (A.C.-U.); pilar.escribano.subias@gmail.com (P.E.-S.); 2 Cardiology Department, Hospital Universitario 12 de Octubre, 28041 Madrid, Spain; juan.duarte@salud.madrid.org (J.D.T.); teresa.segura@salud.madrid.org (T.S.d.l.C.); fernando.arribas@salud.madrid.org (F.A.Y.); 3 Institute for Research, Hospital 12 de Octubre (i+12), 28041 Madrid, Spain; 4 European Reference Network on Rare Respiratory Diseases (ERN-LUNG), 60596 Frankfurt, Germany; 5 Centro de Investigación Biomédica en Red de Enfermedades Cardiovasculares (CIBERCV), 28041 Madrid, Spain; 6 Faculty of Medicine, Complutense University of Madrid, 28040 Madrid, Spain; 7 Pulmonary Hypertension and Heart Failure Division of Instituto de Cardiología de Corrientes, Corrientes 3400, Argentina; marialorecoronel@yahoo.com.ar; 8 Adult Congenital Heart Disease Unit, Department of Cardiology, Hospital Universitario 12 de Octubre, 28041 Madrid, Spain

**Keywords:** pulmonary arterial hypertension, TAPSE/PASP ratio, ventricular–arterial coupling, right heart catheterization, transthoracic echocardiography

## Abstract

**Background/Objectives**: Ventricular-arterial (VA) coupling, assessed via the TAPSE/PASP ratio, is a well-established prognostic marker in pulmonary arterial hypertension (PAH). However, transthoracic echocardiography (TTE) often fails to estimate the pulmonary artery systolic pressure (PASP). This study evaluated the prognostic value of TAPSE/PASP when PSAP was obtained both via TEE and RHC and their correlation. **Methods**: A prospective registry included 90 PAH patients (April 2021–May 2024). TTE and RHC were performed according to clinical guidelines. The correlation and agreement between both techniques were assessed using Spearman’s rank correlation and a Bland–Altman analysis. The prognostic utility of TAPSE/PASP for clinical worsening (CW) (death or lung transplantation) was evaluated using Cox models, Harrell’s c-statistics, and ROC curve analysis. **Results**: The median interval between TTE and RHC was 1.5 days (range −3 to +43). TAPSE/PASP showed a strong correlation between both techniques (rho = 0.74, *p* < 0.001), though TTE slightly overestimated values due to PASP underestimation. The PASP correlation was moderate (rho = 0.56, *p* < 0.001). CW occurred in 17.8% of patients. According to cut-off points established based on ESC/ERS guidelines, VA coupling via TTE effectively stratified the risk of CW (HR 7.0, *p* = 0.076 and HR 34.8, *p* = 0.002 for intermediate and high risk, respectively), whereas VA coupling with PASP measured via RHC showed no association with CW. TAPSE/PASP based on TTE demonstrated superior prognostic performance (C-index = 0.81) over RHC-derived parameters (C-index = 0.58). **Conclusions**: The TAPSE/PASP ratio showed a strong correlation between TTE and RHC. However, while RHC remains the gold standard for hemodynamic assessments, echocardiographic measurements demonstrated superior performance in risk stratification, supporting its role as a valuable non-invasive tool in PAH.

## 1. Introduction

The right ventricular (RV) function is a critical prognostic factor in pulmonary arterial hypertension (PAH). In the early stages of the disease, the RV adapts to the increased afterload by enhancing contractility through compensatory hypertrophy. However, as pulmonary hypertension (PH) progresses, these compensatory mechanisms eventually fail, and contractility can no longer sufficiently increase to match the afterload. This leads to RV dilation, which initially helps maintain the stroke volume through the Frank–Starling mechanism. Over time, this dilation results in a loss of contractile function, creating an imbalance between contractility and the afterload, which marks a deterioration in the patient’s clinical condition and significantly increases the risk of adverse outcomes [1].

In clinical practice, ventricular–arterial (VA) coupling, commonly estimated using the TAPSE/PASP ratio, is the most widely used parameter to assess the relationship between RV contractility and its afterload [2,3]. The prognostic value of this echocardiographic parameter has been extensively validated in various clinical settings, including PAH [4,5,6,7]. Studies have shown that lower TAPSE/PASP values are associated with worse hemodynamic patterns and a higher risk of mortality. Notably, a cutoff of 0.19 was identified as the best threshold for predicting clinical deterioration or death in a cohort of patients with PAH associated with scleroderma [8], while a cutoff of 0.30 was found to be the strongest predictor of death or the need for lung transplantation in a cohort of PAH patients awaiting transplantation [9]. The 2022 ESC/ERS clinical practice guidelines highlight its significance, listing it as one of the three echocardiographic markers recommended for risk stratification in PAH and proposing the following cut-off points: low risk (<0.19 mm/mmHg), intermediate risk (0.19–0.32 mm/mmHg), and high risk (>0.32 mm/mmHg) [10]. These cut-offs are based on the work by Tello et al., who divided a long cohort of PAH patients into TAPSE/PASP tertiles and demonstrated that lower values of this ratio are associated with worse hemodynamic patterns and a higher risk of mortality [4].

While these guidelines reinforce the clinical utility of TAPSE/PASP, its reliability in routine practice remains dependent on the accurate estimation of the pulmonary artery systolic pressure (PASP) via echocardiography. The conventional method, based on the Bernoulli equation, requires the presence of tricuspid regurgitation (TR) with a Doppler spectral recording covering at least 50% of the velocity curve [11]. In clinical practice, approximately 20% of patients lack sufficient TR to obtain a reliable PASP measurement [12,13].

Given the inherent limitations of PASP estimations via TTE, an alternative approach is to derive the TAPSE/PASP ratio using the PASP measured directly via RHC. Since RHC provides invasive hemodynamic measurements, it may offer greater accuracy. However, the prognostic value of RHC-derived TAPSE/PASP compared to its echocardiographic counterpart remains unproven. Therefore, this study aims to assess the correlation between TAPSE/PASP obtained via TTE and RHC and to determine whether RHC-derived TAPSE/PASP provides comparable prognostic significance.

To our knowledge, this is the first study to systematically evaluate this correlation while also assessing the prognostic utility of RHC-derived TAPSE/PASP. By addressing these questions, our findings may contribute to refining risk-stratification strategies and optimizing clinical decision-making in PAH management.

## 2. Materials and Methods

### 2.1. Study Design and Data Collection

From April 2021 to May 2024, consecutive patients from the FIS study “Moving Toward an Omic Classification for Pulmonary Arterial Hypertension” (funding: Instituto de Salud Carlos III, 2022-2024, Project ID: PI21-01690) were included in a prospective registry with a retrospective analysis. A total of 90 patients with PH were enrolled (88 with PAH and 2 with PH associated with neurofibromatosis without lung involvement).

Transthoracic echocardiography (TTE) and RHC were performed, within a short timeframe, without treatment modifications between procedures. TTE was performed according to current guidelines for cardiac chamber quantification [14], and all the parameters for the prognostic evaluation and the PH probability estimation were collected according to the 2022 ESC/ERS guidelines for the diagnosis and treatment of PH [10]. All TTE data were acquired and processed by a cardiac imaging specialist. RHC was performed by certified hemodynamic specialists, and all measurements and data collection were conducted in accordance with the recommendations outlined in the 2022 ESC/ERS guidelines [10]. Clinical data were obtained from the medical records.

The study protocol was approved by the institutional ethics committee (CEIM) at our hospital, and all patients provided written informed consent for the procedures.

### 2.2. Primary and Secondary Endpoints

The primary objectives of this study were as follows:

1. To analyze whether the TAPSE/PASP ratio, with PASP estimated via TTE, correlates with the TAPSE/PASP ratio when PASP is measured via RHC.
2. To evaluate the prognostic impact of TAPSE/PASP, as measured based on both TTE and RHC, in predicting clinical worsening—defined as lung transplantation and/or death—using the risk cutoff values recommended by the ESC/ERS guidelines [10].
3. To assess the prognostic significance of the TAPSE/PASP ratio, as measured via RHC, in the subgroup of patients for whom PASP could not be estimated via TTE.

The secondary endpoints included the following:

1. Comparing PASP measurements obtained via RHC and TTE.
2. Assessing right atrial pressure (RAP) as measured via RHC and estimated via TTE.
3. Investigating whether the accuracy of TTE-derived estimates and their correlations are influenced by the presence of significant TR.

### 2.3. Statistical Analysis

The normality of the data was assessed using the Shapiro–Wilk test, with a *p*-value < 0.05 considered indicative of non-normality. Based on the results of this test, it was determined that the data did not follow a normal distribution. Therefore, non-parametric statistical methods were employed for the analysis.

Categorical data are presented as counts and percentages. Continuous variables are expressed as median values with the interquartile range (IQR).

To evaluate the relationship between RHC and TTE measurements, Spearman’s rank correlation coefficient was calculated, to assess the monotonic relationship between two variables. The agreement between RHC and TTE measurements was evaluated using the Bland–Altman analysis. Statistical significance was determined using a two-tailed *p*-value, with *p* < 0.05 considered statistically significant. To assess the reliability of echocardiographic measurements, the intraclass correlation coefficient (ICC) was calculated to evaluate inter- and intraobserver variability for the TAPSE, PASP, and the TAPSE/PASP ratio. For RHC, inter- and intraobserver variability was not assessed, as this was a real-world study where measurements were taken by the operator during the procedure itself.

Scatter plots were generated to visually assess the relationship between RHC and TTE measurements, displaying the distribution of paired data points.

To evaluate the correlation between RHC and TTE for stratifying patients based on VA coupling, the population was divided into three risk groups according to the ESC/ERS guidelines [10]: low risk (TAPSE/PASP > 0.32), intermediate risk (TAPSE/PASP between 0.19 and 0.32), and high risk (TAPSE/PASP < 0.19) [10]. Kaplan–Meier survival curves were generated to assess differences in survival outcomes across different VA risk categories as measured based on both methods.

The prognostic capacity of the TAPSE/PASP ratio was assessed using Cox regression models to estimate the hazard ratios (HRs) for the composite outcome of death or lung transplantation. The proportional hazards assumption was assessed using the Schoenfeld residual test, and no significant deviations were observed, confirming the validity of the Cox model. Discrimination indices, including the C-Harrel index, Somers’ D, and the area under the receiver operating characteristic curve (AUC ROC), were used to evaluate the predictive performance of the TAPSE/PASP ratio with PASP estimated via TTE and measured via RHC.

The follow-up time was calculated as the difference between the inclusion date and the date of the last follow-up or clinical worsening, including only those patients with a follow-up period exceeding 6 months.

All statistical analyses and graphs were performed with Stata, version 16 (Stata Corp, Lakeway Dr., College Station, TX, USA).

## 3. Results

### 3.1. Patient Characteristics and Risk Stratification

The study included 90 patients, with a median age of 52 years. Most patients were classified as WHO Functional Class II (48.9%), followed by Class III (31.1%). The most common etiology of PAH was idiopathic (35.6%), followed by associated with connective tissue disease (17.8%).

Hemodynamically, the median mean pulmonary artery pressure (mPAP) was 48 (37–55) mmHg, cardiac output was 4.6 (3.7–5.5) L/min, and pulmonary vascular resistance (PVR) was 7.9 (5.7–11.5) WU.

For the echocardiographic parameter, the median PASP was 72 (52–93) mmHg, the median TAPSE was 19 (16–21) mm, and the median TAPSE/PASP ratio was 0.24 (0.18–0.38) mm/mmHg. The interobserver and intraobserver variability of echocardiographic measurements are shown in Supplementary Table S1.

Regarding treatment, most patients were on dual oral therapy (41.1%), followed by triple therapy with parenteral prostacyclin (27.8%). Table 1 summarizes the baseline data.

The median time between RHC and TTE was 1.5 days (range −3 to +43 days). The median follow-up time was 24 months (14–35 months). Of the 90 patients included, 30.0% (n = 27) lacked a sufficient TR to estimate the PASP via TTE.

### 3.2. Hemodynamic Parameter Correlations with Transthoracic Echocardiography

When analyzing the correlation between TAPSE/PASP measured via RHC and TTE in the 63 patients eligible for estimating the PASP via TTE, the results demonstrated a strong positive correlation (rho = 0.74, *p* < 0.0001). The Bland–Altman analysis revealed a mean bias of 0.04 (95% CI 0.01–0.06) between TAPSE/PASP via TTE and RHC, with slightly higher values observed via TTE due to an underestimation of the PASP. The limits of agreement ranged from −0.17 to 0.24, encompassing 95% of the differences. Lin’s concordance correlation coefficient was 0.60, indicating moderate agreement. Proportional bias was not significant.

Correlations between the remaining hemodynamic parameters and their estimates based on TTE revealed the following:

1. PASP: A moderate positive correlation was found (rho = 0.56, *p* < 0.0001). The Bland–Altman analysis revealed a mean bias of −7.99 mmHg (95% CI −13.75 to −2.24), with TTE generally underestimating the PASP compared to RHC. Limits of agreement ranged from −52.76 to 36.77 mmHg, indicating substantial variability in individual measurements. Lin’s concordance correlation coefficient was 0.53, reflecting moderate agreement. No evidence of proportional bias was observed.
2. RAP: The correlation was weak and not statistically significant (rho = 0.22, *p* = 0.0857). The Bland–Altman analysis comparing RAP revealed a mean bias of −2.03 mmHg (95% CI: −3.36 to −0.70), indicating a tendency for the underestimation of RAP via TTE. Limits of agreement ranged from −12.23 mmHg to 8.23, demonstrating considerable variability between methods. Lin’s concordance correlation coefficient was 0.36, reflecting limited agreement. No evidence of proportional bias was observed.

**Figure 1:**
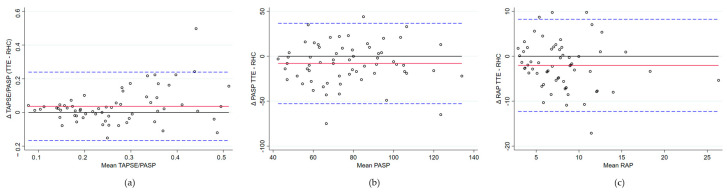
Bland–Altman analysis of the correlations between hemodynamic parameters measured via right heart catheterization (RHC) and transthoracic echocardiography (TTE). (**a**) TAPSE/PASP, (**b**) Pulmonary artery systolic pressure (PASP). (**c**) Right atrial pressure (RAP). Red line represents the mean difference between the two measurement methods. Blue dashed lines indicate the limits of agreement (±1.96 standard deviations).

Figure 1 and Table 2 show the analysis of these correlations.

### 3.3. Stratified Analysis Based on Significant Tricuspid Regurgitation

Twelve patients (13.3%) had significant TR (defined as higher than moderate TR) on TTE. The stratified analysis based on the presence of significant TR showed notable differences in correlations.

1. The TAPSE/PASP ratio showed a moderate correlation (rho = 0.65, *p* < 0.0001) for patients without significant TR. In patients with significant TR, the correlation was notably higher (rho = 0.82, *p* = 0.0012), indicating a strong and significant relationship between the two parameters.
2. For the PASP, a moderate correlation was found in patients without significant TR (rho = 0.46, *p* = 0.0006), suggesting a significant positive relationship. In patients with significant TR, the correlation tended to be slightly stronger, although still moderate (rho = 0.54), but was marginally non-significant (*p* = 0.0676).
3. No significant correlation was found between RAP estimated via TTE and measured via RHC, either in patients without significant TR (rho = 0.10, *p* = 0.5040) or in those with significant TR (rho = 0.48, *p* = 0.1137), indicating a lack of relationship between the two parameters.

**Figure 2:**
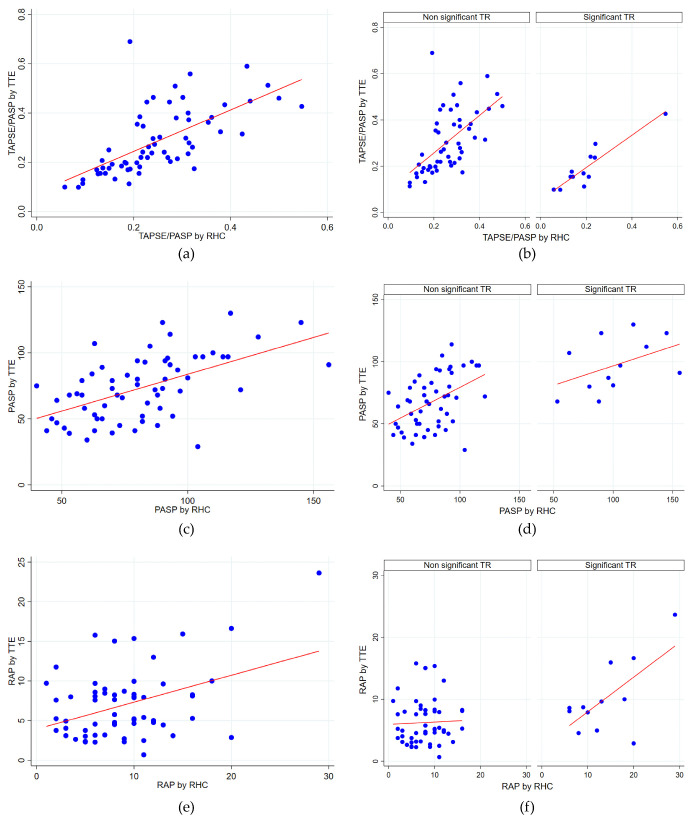
Relation between different hemodynamic parameters measured via RHC and estimated via TTE. (**a**) TAPSE/PASP correlation in all patients (**b**) TAPSE/PASP correlation in patients stratified by significant TR. (**c**) PASP correlation in all patients (**d**) PASP correlation in patients stratified by significant TR. (**e**) RAP correlation in all patients (**f**) RAP correlation in patients stratified by significant TR. PASP: pulmonary artery systolic pressure; RAP: right atrial pressure; RHC: right heart catheterization; TAPSE: tricuspid annular plane systolic excursion; TR: tricuspid regurgitation; TTE: transthoracic echocardiography.

Figure 2 shows the scatter plots illustrating the correlations between the measured parameters.

### 3.4. Prognostic and Predictive Value of TAPSE/PASP via Echocardiography and Right Heart Catheterization

During the follow-up period, 16 patients (17.8%) experienced clinical worsening, 11 patients (12.2%) died, and 5 (5.6%) underwent lung transplantation.

When stratifying patients according to the REVEAL Lite 2.0 Score [15], 56 patients (62.2%) were classified as low risk, 20 patients (22.2%) as intermediate risk, and 14 patients (15.6%) as high risk. The REVEAL Lite 2.0 score demonstrated appropriate risk stratification, with increasing hazard ratios corresponding to higher risk categories: for the intermediate risk group, HR = 8.07 (*p* = 0.001), and for the high-risk group, HR = 11.48 (*p* = 0.001), reflecting a significantly higher risk of adverse events. The predictive performance of the REVEAL Lite 2.0 score was further supported by a Harrell’s C of 0.70 (0.56–0.84), Somers’ D of 0.41, and an AUC of 0.73 (0.60–0.86).

Regarding the prognostic impact of TAPSE/PASP, when stratifying patients according to the ESC/ERS guidelines [10] cut-off values for VA coupling, we found:

1. For TAPSE/PASP estimated via TTE (N = 63): 22 patients (34.9%) were at low risk, 23 patients (36.5%) at intermediate risk, and 18 patients (28.6%) at high risk. VA coupling measured via TTE showed appropriate risk stratification with an increasing HR as the risk category rose. For the intermediate risk group, HR = 7.03 (*p* = 0.076) and for the high-risk group, HR = 34.81 (*p* = 0.002). The Harrell’s C statistic was 0.81 (0.73–0.89), indicating good predictive ability, while the Somers’ D statistic was 0.62, suggesting a positive association between predictions and observed events.
2. For TAPSE/PASP estimated via RHC in those patients with PASP estimated via TTE (N = 63): 12 patients (19.1%) were at low risk, 33 patients (52.4%) at intermediate risk, and 18 patients (28.6%) at high risk. VA coupling using PASP measured via RHC did not show a relationship with clinical worsening (HR 1.13, *p* = 0.877 for intermediate risk and HR 2.3, *p* = 0.315 for high risk). The Harrell’s C concordance statistic for the model was 0.59 (0.46–0.71) suggesting limited predictive ability, while Somers’ D statistic was 0.17 suggesting a low association with the events.
3. When analyzing all patients, including those without PASP estimated via TTE (N = 90), no relationship with clinical worsening was found either.

**Figure 3:**
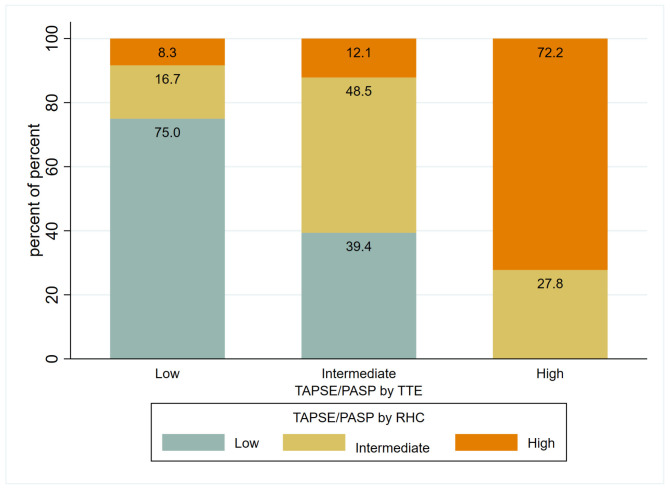
Distribution of risk categories (low, intermediate, high) based on TAPSE/PASP values measured via TTE and RHC, using cut-offs from the ESC/ERS 2022 guidelines. Low risk: TAPSE/PASP > 0.32, intermediate risk: TAPSE/PASP 0.19–0.32, and high risk: TAPSE/PASP < 0.19. ESC/ERS, European Society of Cardiology/European Respiratory Society, PASP, pulmonary artery systolic pressure; RHC, right heart catheterization; TAPSE, tricuspid annular plane systolic excursion; TTE, transthoracic echocardiography.

Figure 3 shows the redistribution of patients into different categories based on the stratification using the cutoff points of the TAPSE/PASP ratio based on TTE and RHC guidelines. Table 3 presents the results of the analysis of the prognostic value of TAPSE/PASP using both techniques. The results for additional exploratory cut-off points of TAPSE/PASP are presented in Supplementary Table S2.

For PASP estimated via TTE, the analysis revealed higher values observed in patients who died or were transplanted compared to those who did not suffer any event (z = −2.737, *p* = 0.0062). Conversely, when measured via RHC, there was no significant difference (z = −0.565, *p* = 0.5722). The maximum TR velocity based on Doppler showed also a significant difference (z = −2.686, *p* = 0.0072), with higher velocities in the group of patients who presented the event. For RAP via echo and via RHC, there were no significant differences between both groups (z = −0.646, *p* = 0.5182 and z = −1.682, *p* = 0.0926, respectively). The TAPSE was significantly lower in those patients who experienced the event (z = 2.43, *p* = 0.0150).

**Figure 4:**
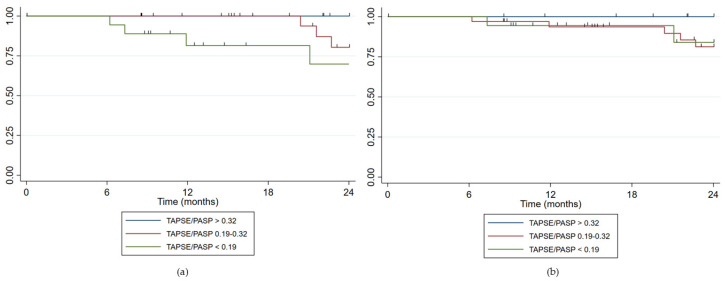
Survival curves stratified according the TAPSE/PASP cut-off points of ESC/ERS guidelines. (**a**) PASP estimated via TTE; (**b**) PASP measured via RHC.

Figure 4 shows the survival curves stratified according the TAPSE/PASP cut-off points of ESC/ERS guidelines [10] based on TTE and RHC.

### 3.5. Subgroup Analysis of Patients with Non-Estimable PASP via Echocardiography

In the subgroup of patients without PASP estimable via TTE (30%, N = 27), the TAPSE/PASP ratio measured via RHC was not associated with the combined event of death or transplantation (*p* = 0.802).

Similarly, when stratified using the TAPSE/PASP cut-off values proposed by the ESC/ERS guidelines [8], the Cox analysis showed no significant association with clinical worsening. Specifically, no association was found in either risk group: intermediate-risk (HR = 11.49, *p* = 0.084) and high-risk (HR = 3.35 × 10^−16^, *p* = 1.000). These results are likely influenced by the very low prevalence of events in this subgroup (2 out of 27 patients, 7.41%).

## 4. Discussion

The main findings of this study can be summarized as follows: (1) the TAPSE/PASP ratio measured via both TTE and RHC demonstrated a strong correlation, underscoring its reliability across both diagnostic modalities; (2) the echocardiographic estimation of PASP showed moderate agreement with RHC, with a tendency toward underestimation and notable variability between methods; (3) the presence of significant TR improved the correlation of PASP measurements obtained using both techniques; and (4) despite an adequate correlation, the prognostic value of the TAPSE/PASP ratio derived from echocardiography was not observed when PASP was measured invasively via RHC.

Since Tello et al. validated the TAPSE/PASP ratio as a non-invasive measure of VA coupling, and this parameter has gained significant attention in the assessment of PAH [2]. VA decoupling, as reflected by a decrease in the TAPSE/PASP ratio, marks a critical transition point toward maladaptive remodeling and has been shown to carry prognostic significance in PAH. Numerous studies have corroborated this prognostic impact and proposed various cut-off values for risk stratification. For instance, Tello et al. stratified patients by tertiles, establishing cutoff values of 0.19 and 0.32 in their study population to divide patients into three distinct risk groups [4]. These cutoff values were subsequently incorporated into the 2022 ESC/ERS guidelines for PH, where the TAPSE/PASP ratio was included as one of three echocardiographic parameters in a three-strata risk stratification system [10]. Recently, Ghio et al. proposed a right ventricular phenotyping approach in incident patients with idiopathic PAH, using a cut-off value of 0.33 for the TAPSE/PASP ratio to categorize patients into distinct risk phenotypes [16]. This further highlights the utility of the TAPSE/PASP ratio in characterizing ventricular function and prognosis in PH.

A significant limitation of TTE in the assessment of VA coupling is that not all patients have measurable PASP values due to insufficient TR, which excludes a subset of individuals from benefiting from this prognostic parameter. It is estimated that approximately 20% of patients in general cohorts lack sufficient TR to allow for an accurate PASP estimation, as reported in some large patient cohorts [12,17]. Previous studies examining the correlation between echocardiography and hemodynamics in PH have reported that approximately 10% of patients lack measurable TR [18,19,20,21,22]. However, these findings have not been consistent across all groups of patients; for example, Denton et al. observed a higher proportion (39%) in systemic sclerosis patients with suspected PH [23].

In our study, the feasibility of PASP estimations via echocardiography was slightly lower (70%) compared to prior reports. This discrepancy may be attributed to the high proportion of patients in a low-risk clinical scenario, characterized by minimal ventricular dilation (median RV/LV ratio of 1.1), which could reduce the likelihood of detectable TR. Regardless of the variability in the percentage of patients without estimable PASP, TAPSE/PASP is a very useful parameter that should be available in all patients.

Given that all patients undergo RHC to confirm the diagnosis of PH and that many of them return for a hemodynamic reassessment during disease follow-up, we proposed that the TAPSE/PASP ratio, using direct measurements of PASP via catheterization, could help estimate VA coupling in this subgroup of patients.

Multiple studies have explored the correlation between echocardiographic and hemodynamic parameters in PH, showing some variability in the strength of these correlations, mostly ranging from a correlation coefficient of 0.6 to 0.8 (moderate-to-strong correlation). This variability can be influenced by several factors. Data from the REVEAL registry found that the correlation between PASP measurements obtained from catheterization and echocardiography was stronger when both tests were performed on the same day (rho = 0.76) compared to when they were conducted within the same year (rho = 0.56), suggesting that the accuracy of echocardiographic PASP estimation diminishes over time [24]. In contrast, other reports showed only moderate correlations in cohorts where both techniques were performed within 24–72 h [18,21,22]. Additional factors have been proposed to contribute to the variability and they include the female gender, arrhythmic cardiac electrical activity, systemic arterial hypertension, and diuretic treatment [21].

In our study population, we found a moderate positive correlation for PASP (rho = 0.56) in the overall cohort, which is comparable to the correlation observed in the REVEAL registry in which both tests were not performed simultaneously. When assessing TAPSE/PASP, the correlation improved, reaching a strong positive correlation (rho = 0.74) between RHC and TTE-derived measurements, and was even higher (rho = 0.82) in patients with significant TR.

Although a strong correlation between both measures of VA coupling was found, the parameter estimated based on catheterization failed to demonstrate prognostic value. This discrepancy raises important questions about the underlying mechanisms and potential confounding factors that may influence the prognostic utility of this parameter. One potential explanation for this finding could be the asymmetric distribution of error in the PASP estimation, with a tendency to overestimate higher PASP values measured based on catheterization.

Even though TAPSE/PASP measured via echocardiography has been extensively validated for prognostic stratification in previous studies, to the best of our knowledge, this is the first study to propose validating this parameter using the invasively measured PASP. Despite the observed correlation, our findings did not confirm its prognostic utility, highlighting the need for further research to better understand the differences between these methodologies and their clinical implications.

Future studies with larger sample sizes, longer follow-up periods, and standardized protocols for PASP measurements are warranted to clarify these findings and explore potential applications in risk stratification.

### Limitations

This is a retrospective, observational, single-center study, which may limit the generalizability of the results. The sample size may not be large enough to detect subtle differences or to apply the findings to broader populations. Additionally, the limited number of patients and events prevented the use of a multivariable Cox regression analysis, hindering our ability to assess potential confounding factors. Furthermore, the subgroups of patients with significant TR and those without estimable PASP were relatively small, which may have reduced the statistical power to identify meaningful differences and limited the relevance of the conclusions drawn.

Second, as an observational study, inherent biases and confounding factors cannot be completely ruled out. Although no changes in treatment occurred between RHC and TTE, the time interval between both procedures (TTE and RHC) may have influenced the observed differences in measurements.

Lastly, this study was conducted under real-world conditions, without the standardized repetition of measurements. While this enhances external validity, it may have introduced variability in the data-collection process.

## 5. Conclusions

Our findings demonstrate a strong correlation between the TAPSE/PASP ratio measured via TTE and RHC, confirming its potential for assessing VA coupling in patients with PAH. However, despite this correlation, the TAPSE/PASP ratio calculated using invasive PASP did not prove to be a reliable prognostic predictor, even though RHC remains the gold standard for hemodynamic assessments. These findings suggest that TAPSE/PASP ratio clinical utility may depend on the method used to obtain the PASP.

The quality of an echocardiographic measurement—partially influenced by the presence of TR—remains a significant limitation, as PASP could not be estimated via TTE in a substantial proportion of patients. This highlights the need to optimize non-invasive assessment strategies to improve the diagnostic accuracy and risk stratification in this population.

Given the prognostic value demonstrated based on the TAPSE/PASP ratio obtained through TTE, future studies are needed to address the knowledge gap caused by the lack of the TAPSE/PASP ratio in patients without sufficient TR.

## Figures and Tables

**Table 1:** Baseline characteristics. 6MWD: 6-min walking distance; BMI: body mass index; IPAH: idiopathic pulmonary arterial hypertension; LV: left ventricle;; PVOD: pulmonary veno-occlusive disease; RA: right atria; REVEAL: Registry to Evaluate Early and Long-Term PAH Disease Management; RV: right ventricle; TAPSE: tricuspid annular plane systolic excursion; TR: tricuspid regurgitation; WHO-FC: World Health Organization Functional Class.

Demographic Characteristics	N = 90
Age, y	52 (42–63)
Female/male	2.5/1
BMI, kg/m^2^	26.0 (22.5–30.0)
Disease characteristics at baseline	
WHO-FC	
Functional Class I	16 (17.8)
Functional Class II	44 (48.9)
Functional Class III	28 (31.1)
Functional Class IV	2 (2.2)
6MWD, m	411 (322–495)
REVEAL Lite risk score	
≤6	56 (62.2)
7–8	20 (22.2)
≥9	14 (15.6)
Time from diagnoses (months)	2.6 (4.0–8.4)
PAH pathogenesis	
Group 1	
IPAH	32 (35.6)
Heritable	12 (13.3)
Drugs and toxins	4 (4.4)
Associated with	
Connective tissue disease	16 (17.8)
Portal hypertension	1 (1.1)
Congenital heart disease	8 (8.9)
PVOD	15 (16.7)
Group 5 (neurofibromatosis)	2 (2.1)
Hemodynamic measures	
Systolic pulmonary artery pressure, mmHg	73 (61–91)
Diastolic pulmonary artery pressure, mmHg	30 (23–37)
Mean pulmonary artery pressure, mmHg	48 (37–55)
Pulmonary arterial wedge pressure, mmHg	10 (8–12)
Cardiac output, l/min	4.6 (3.7–5.5)
Cardiac index, l/minxm^2^	2.7 (2.2–3.1)
Pulmonary vascular resistance, WU	7.9 (5.7–11.5)
Right atrial pressure, mmHg	8 (5–10)
Echocardiographic data	
Right atria area (cm^2^)	19 (17–24)
Systolic pulmonary artery pressure, mmHg	72 (52–93)
TAPSE (mm)	19 (16–21)
TAPSE/PASP (mm/mmHg)	0.24 (0.18–0.38)
RV/LV ratio	1.1 (0.9–1.2)
Eccentricity diastolic index	1.3 (1.1–1.5)
Pericardial effusion (presence)	13 (14.4)
Estimated RA pressure (mmHg)	5 (4–9)
Grade TR	
None	27 (30.0))
Mild	30 (33.3)
Mild to moderate	21 (23.3)
Moderate to severe	6 (6.7)
Severe	6 (6.7)
Treatment strategy	
Single therapy	6 (6.7)
Dual therapy	37 (41.1)
Triple oral therapy	10 (11.1)
Oral therapy with systemic prostanoid	11 (12.2)
Triple parenteral therapy	25 (27.8)

**Table 2:** Bland–Altman analysis and Spearman’s rank correlation analysis for TAPSE/PASP, PASP, and RAP measured via transthoracic echocardiography and right heart catheterization. CCC: concordance correlation coefficient; CI: confidence interval; PSAP: pulmonary artery systolic pressure; RAP: right atrial pressure; TAPSE: tricuspid annular plane systolic excursion.

	Bland–Altman Analysis			Spearman’s Rank Correlation	
	Bias	95% CI	Lin’s CCC	Coefficients	*p* Values
TAPSE/PASP	0.04	0.01–0.06	0.60	0.74	<0.0001
PASP	−7.99	−13.75–−2.24	0.53	0.56	<0.0001
RAP	−2.03	−3.36–−0.70	0.36	0. 22	0.0857

**Table 3:** Patient classification based on TAPSE/PASP cut-offs from ERS/ESC guidelines, prevalence of death or lung transplantation across risk groups, Cox regression analysis, discrimination and predictive performance metrics. AUC ROC: area under the curve, receiver operating characteristic; HR: hazard ratio; PSAP: pulmonary artery systolic pressure; TAPSE: tricuspid annular plane systolic excursion.

	n (%)	Death or Lung Transplantion n (%)	Cox Regression HR (*p* Value)	Discrimination Indices Predictive Performance
TAPSE/PASP with PASP estimated by TTE (N = 63)				
TAPSE/PASP > 0.32	22 (34.9)	1 (1.6)	--	C-Harrel: 0.81 (0.73–0.89) Somers’ D: 0.62 AUC ROC: 0.75 (0.62–0.85)
TAPSE/PASP 0.19–0.32	23 (36.5)	5 (7.9)	HR 7.03 (0.076)	
TAPSE/PASP < 0.19	18 (28.6)	8 (12.7)	HR 34.81 (0.002)	
TAPSE/PASP with PASP measured by RHC in patients with PASP estimable by TTE (N = 63)				
TAPSE/PASP > 0.32	12 (19.1)	2 (3.2)	--	C-Harrel: 0.58 (0.46–0.71) Somers’ D: 0.17 AUC ROC: 0.56 (0.43–0.68)
TAPSE/PASP 0.19–0.32	33 (52.4)	7 (11.1)	HR 1.13 (0.877)	
TAPSE/PASP < 0.19	18 (28.6)	5 (7.9)	HR 2.33 (0.315)	
TAPSE/PASP with PASP measured by RHC (N = 90)				
TAPSE/PASP > 0.32	23 (25.6)	3 (3.3)	--	C Harrel: 0.57 (0.43–0.71) Somers’ D: 0.14 TAUC ROC: 0.56 (0.45–0.67)
TAPSE/PASP 0.19–0.32	45 (50.0)	8 (8.8)	HR 1.52 (0.547)	
TAPSE/PASP < 0.19	22 (24.4)	5 (5.5)	HR 2.60 (0.193)	

## Data Availability

The data supporting the findings of this study are available from the corresponding author upon reasonable request.

## Supplementary Materials

The following supporting information can be downloaded at: https://www.mdpi.com/article/10.3390/jcm14061968/s1, Table S1: Interobserver and Intraobserver Variability of Echocardiographic Measurements; Table S2: Predictive Values of TAPSE/PASP Based on Various Cut-off Points: Hazard Ratios and Statistical Significance.

## Abbreviations

The following abbreviations are used in this manuscript:

C-statistics	Harrell’s C-statistics (a measure of model discrimination)
CI	Confidence Interval
C-index	Concordance Index
CW	Clinical Worsening
ESC/ERS	European Society of Cardiology/European Respiratory Society
HR	Hazard Ratio
IQR	Interquartile Range
Lin’s CCC	Lin’s Concordance Correlation Coefficient
mPAP	Mean Pulmonary Artery Pressure
PASP	Pulmonary Artery Systolic Pressure
PH	Pulmonary Hypertension
PVR	Pulmonary Vascular Resistance
RAP	Right Atrial Pressure
RHC	Right Heart Catheterization
REVEAL Lite 2.0	Registry to Evaluate Early and Long-Term PAH Disease Management (REVEAL) abridged version risk calculator.
Spearman’s rho	Spearman’s Rank Correlation Coefficient
TAPSE	Tricuspid Annular Plane Systolic Excursion
TTE	Transthoracic Echocardiography
TR	Tricuspid Regurgitation
VA	Ventricular-arterial
WHO	World Health Organization
