# Supplementary material for: Comparison of Tricuspid Annular Plane Systolic Excursion to Pulmonary Artery Systolic Pressure Ratio Measured by Transthoracic Echocardiography and Right Heart Catheterization in Pulmonary Arterial Hypertension: Prognostic Implications
Source: J Clin Med. 2025 Mar 14;14(6):1968. doi: 10.3390/jcm14061968 (PMC11942982; doi:10.3390/jcm14061968)
Supplement: Supplementary file 1 [file jcm-14-01968-s001.zip › jcm-3496950-supplementary.pdf]

Supplementary Table 1: Interobserver and Intraobserver Variability of Echocardiographic Measurements

Variable	Type of Variability	ICC (Individual)	95% CI (Individual)	ICC (Average)	95% CI (Average)	p-value
**TAPSE**	Interobserver	0.956	0.874–0.985	0.977	0.933–0.992	<0.001
	Intraobserver	0.959	0.882–0.986	0.979	0.937–0.993	<0.001
**PASP**	Interobserver	0.976	0.927–0.992	0.988	0.962–0.996	<0.001
	Intraobserver	0.977	0.931–0.993	0.989	0.964–0.996	<0.001
**TAPSE/ PASP Ratio**	Interobserver	0.971	0.913–0.991	0.985	0.954–0.995	<0.001
	Intraobserver	0.957	0.872–0.986	0.978	0.931–0.993	<0.001

Supplementary Table 2: Predictive Values of TAPSE/PASP Based on Various Cut-off Points: Hazard Ratios and Statistical Significance

Cut-off Criteria	Method	Cut-off	HR (95% CI)	p-value
**Optimal ROC-based cut-off**	TTE	0.17	4.16 (1.09-15.85)	0.037
	RHC	0.27	1.93 (0.70-5.35)	0.204
**Tertile-based cut-offs**	RHC	Tertile 1 (< 0.21)	Reference	-
		Tertile 2 (0.21-0.30)	1.81 (0.43-7.59)	0.416
		Tertile 3 (>0.30)	4.37 (1.15-16.63)	0.031
	TTE	Tertile 1 (< 0.20)	Reference	
		Tertile 2 (0.20-0.32)	6.44 (0.72-57.81)	0.096
		Tertile 3 (>0.32)	25.81 (3.00-221.89)	0.003
**Dichotomous cut-off = 0.19**	RHC	0.19	1.95 (0.67-5.64)	0.218
	TTE	0.19	9.45 (2.75-32.47)	0.000
**Dichotomous cut-off = 0.32**	RHC	0.32	1.80 (0.51-6.34)	0.358
	TTE	0.32	12.35 (1.60-95.35)	0.016
